# Supplemental Bee Pollen Ingestion With Resultant Anaphylaxis and Allergic Asthma: Case Report

**DOI:** 10.1155/crii/9583906

**Published:** 2026-06-26

**Authors:** Erin Eickholt, Victoria Norelli, Kathleen Martin, Marina Diioia

**Affiliations:** ^1^ Clinical Sciences Department, West Virginia School of Osteopathic Medicine, Lewisburg, WV, USA, wvsom.edu; ^2^ Biomedical Sciences Department, West Virginia School of Osteopathic Medicine, Lewisburg, WV, USA, wvsom.edu

**Keywords:** allergic asthma, anaphylaxis, case report, immunology, pollen

## Abstract

Bee pollen has become an increasingly popular dietary supplement due to potential therapeutic effects. However, ingestion has been shown to trigger allergic responses as severe as anaphylaxis. This case highlights allergic asthma development in a 26‐year‐old female patient with subsequent anaphylaxis secondary to bee pollen ingestion. This patient presented to the emergency department with complaints of lip and tongue swelling, dyspnea, nausea, abdominal pain, and itching 4 h after bee pollen ingestion. Her medical history included seasonal allergic rhinitis and prior exercise‐induced asthma that had not required treatment for 10 years. She was treated with a standard anaphylaxis pharmacological regimen and discharged. The patient then began to experience dyspnea and wheezing 1 month later during peak fall pollen levels. On allergy testing, the patient’s total serum immunoglobulin E (IgE) was elevated at 189 kU/L. She also had elevated specific IgE to common ragweed, mugwort, and cocklebur, which are prominent allergens in the fall. Given the timing of onset of new symptoms, partial relief with an inhaled short‐acting B2 agonist, and no pertinent findings for other causes, the patient was subsequently diagnosed with allergic asthma secondary to bee pollen ingestion. Although anaphylaxis to bee pollen is rare, it is becoming increasingly reported in literature and demonstrates an adverse effect that can occur when proper regulation and education are not performed. Dietary supplements like bee pollen need to have further testing and contain more thorough warnings on labels to decrease adverse events and protect people from undue harm.

## 1. Introduction

Bee pollen is a nutritious product of honey bees that is composed of pollen grains, salivary secretions of the bees, and nectar, as well as many bioactive micronutrients [1]. The exact composition of bee pollen can vary based on the season, location, and flower origin [1]. It has garnered increasing interest as a dietary supplement due to the potential therapeutic effects seen in medical management as well as anti‐inflammatory and anticancer properties [2, 3]. It has also been found, however, that bee pollen can contain harmful ingredients, including environmental toxins such as pesticides and heavy metals. Additionally, bee pollen can trigger allergic responses through exposure to bee pollen via ingestion, sensitization, or cross‐reactivity of aeroallergens [4]. Consumption of bee pollen has increased in prevalence over the years as a result of its potential therapeutic effects. Bee pollen has become a common addition in a variety of health foods and can also be found in foods through cross‐contamination. In comparison, sensitization and cross‐sensitization of aeroallergens can occur unknowingly. Sensitization is the process by which an allergen‐specific immunoglobulin E (IgE) develops [5]. Once the allergen makes direct contact with the skin, respiratory tract, or gastrointestinal tract, the antigen‐presenting cells (APCs) induce the differentiation of naive T‐cells into Th2 cells. The Th2 cells in turn secrete two cytokines, interleukin‐4 (IL‐4) and IL‐13, that influence B‐cells to undergo class switching into the allergen‐specific IgE [5]. Sensitization, specifically to pollen aeroallergens, can take time to develop but has been shown in children as young as 2‐years old and continues to increase with age [6]. In comparison to sensitization, cross‐reactivity occurs when IgE antibodies developed for one specific allergen are also induced to another allergen that shares a similar molecular structure [7]. This can be observed in individuals that have an allergy to a specific aeroallergen pollen that experience an allergic event when consuming food from a plant that has similar molecular components to that of the sensitized pollen [7].

While allergic responses and hypersensitivities are rare, they can vary from benign responses to those more severe, including anaphylaxis [8].

In recent years, there have been increasingly documented reports of allergic responses to bee products. However, there has been less documentation of subsequent allergic asthma development postanaphylaxis secondary to bee pollen ingestion. Allergic asthma is defined as a type of chronic inflammatory airway disease that is triggered by the inhalation of allergens, resulting in an allergic reaction [4]. These triggering substances that one’s immune system mistakes as harmful can include mold, pollen, dust, etc. Once inhaled, the immune system mounts a response against these substances, including the release of mast cells and eosinophils, bronchoconstriction, and increased mucus production that can result in difficulty breathing, wheezing, or chest tightness in affected individuals. Here we present a rare case of subsequent allergic asthma development in a 26‐year‐old female patient with subsequent anaphylaxis secondary to bee pollen ingestion.

## 2. Case Presentation

The patient is a 26‐year‐old female who presented to the Emergency Department with lip and tongue swelling 4 h after ingestion of bee pollen from a supermarket. She denied known food or drug allergies but endorsed a history of seasonal allergic rhinitis in autumn and spring. The patient reported, immediately after ingesting the bee pollen, she had abdominal pain and nausea and felt “itchy all over” without a corresponding rash. The patient also endorsed shortness of breath. On physical examination, the patient was anxious appearing while seated on the exam table. Upper lip swelling and tongue swelling were noted. Vital signs on presentation were as follows: 114/78 mm/Hg, HR 96 bpm, respiratory rate 20 breaths/min, and temperature 36.7°C. Her symptoms were treated with one IM epinephrine injection, IV famotidine histamine −2 antagonist, diphenhydramine histamine‐1 antagonist, IV corticosteroid, and one normal saline bolus. She was discharged after 4 h of observation with diagnosis of angioedema of the lips without return of symptoms with a prescription for 6 days of oral corticosteroid taper and an epinephrine autoinjector.

The patient presented to urgent care approximately 1 month later at the start of fall allergy season with complaints of dyspnea with wheezing. She was prescribed an albuterol inhaler. The patient presented to her primary care provider several times after initiation of the albuterol inhaler with full compliance for continued dyspnea. No wheezes were heard in the patient’s lungs during the visits, suggesting partial improvement with the albuterol inhaler. Without complete symptomatic relief, the patient was referred to a pulmonologist. After referral, an unremarkable chest X‐ray, spirometry, and DLCO, pre‐ and post‐bronchodilator all unremarkable, and normal levels of D‐dimer and alpha‐1 antitrypsin, and elevated levels of IgE and continued dyspnea, she was diagnosed with asthma. The patient only had a pertinent prior history of exercise‐induced asthma, which was asymptomatic and did not require pharmacological therapy for the past 10 years.

In subsequent serum allergy testing 3 months after the inciting incident, the patient’s total serum IgE was elevated at 189 kU/L. As shown in Table 1, she also had specific IgE to common ragweed 23.00 kU/L (Class IV: Very High), mugwort 9.15 kU/L (Class III: High), and cocklebur 9.38 kU/L (Class III: High). Upon a skin prick test, the patient again showed sensitivity to ragweed, mugwort, and cocklebur. She is currently maintained on fluticasone/salmeterol, a combined inhaled corticosteroid/long‐acting beta‐2 agonist; an albuterol rescue inhaler; cetirizine, a histamine‐1 antagonist; and azelastine/fluticasone, an antihistamine/steroid intranasal spray. The patient also elected to begin allergen immunotherapy.

**Table 1:** Patient’s IgE serum test results.

Allergen	IgE serum level	Skin prick testing	Seasonal activity
Common ragweed	23.00 kU/L Class IV; very high	Positive	Autumn (August–November)
Cocklebur	9.38 kU/L Class III; high	Positive	Autumn (August–November)
Mugwort	9.15 kU/L Class III; high	Positive	Autumn (August–November)
Sheep sorrel	0.82 kU/L Class II; moderate	Positive	Autumn (August–November)
Rough pigweed	0.61 kU/L Class I; low	Positive	Autumn (August–November)

## 3. Discussion

This patient, to our knowledge, is the first reported case of new‐onset allergic asthma secondary to anaphylaxis from bee pollen ingestion. At least 13 other cases of anaphylaxis from bee pollen ingestion have been reported, and almost all of the cases, as with this patient, report a history of allergic rhinitis [9]. Despite bee pollen containing honey, wax, and saliva in addition to the pollen from plants, the likely factor causing the allergic reactions is pollen granules [10]. As mentioned, all previous reported cases of anaphylactic reactions to bee pollen ingestion occurred in individuals who had a past medical history of seasonal allergic rhinitis. This knowledge demonstrates that these individuals were presensitized to the pollen prior to their anaphylactic reactions. This conclusion is drawn from the evidence that during the seasonal allergic rhinitis, these individuals were already having less severe allergic reactions to the pollen through premade IgE molecules degranulating mast cells that manifested in typical allergic rhinitis symptoms such as rhinorrhea and sneezing [9]. Thus, when these individuals ingested a large quantity of pollen, the preexisting IgE triggered systemic mast cell degranulation, resulting in an anaphylactic reaction. In previous reports of reactions to bee pollen, one major culprit found was pollen from the Compositae family [10]. This family contains ragweed, mugwort, and cocklebur, three types of pollen our patient tested positive for during allergy testing, as seen in Table 1 [11]. Ragweed, cocklebur, and mugwort pollen are highly prevalent across the United States, with levels peaking from August to September in the Eastern U.S [12]. This demographic and timeline matches our patient’s previous exposure to and episodes of fall allergic rhinitis as well as her subsequent onset of allergic asthma.

This diagnosis was further strengthened after the patient received serum and skin prick allergy testing, which showed that the patient was highly allergic to ragweed, mugwort, and cocklebur pollens, pollens commonly found in bee pollen supplements and known to cause seasonal allergic rhinitis [9].

Bee‐pollen‐induced anaphylactic incidents are being reported more frequently in the literature. However, with the popularity of the supplement growing through social media platforms, it is essential for additional research and data to be published about the harmful side effects of the supplement.

## 4. Conclusion

In conclusion, this is the first known case of new‐onset allergic asthma secondary to bee‐pollen‐induced anaphylaxis. This patient had a history of seasonal allergic rhinitis that indicated a preexisting formation of serum IgE to pollen granules. Upon ingestion of the pollen, the preformed IgE triggered systemic mast cell degranulation, resulting in anaphylaxis. After the reaction, it is suspected that there was an increase in the sensitivity to these allergens, as demonstrated by the patient’s worsening seasonal symptoms and new‐onset allergic asthma.

Further research should be done and supplements should be tested and required to contain warning labels about adverse effects before being sold and advertised to the general population. This will help deter cases like this one, which are preventable and may cause residual harm to patients who are trying to better their health with supplements or other natural products.

## Funding

No funding was received for this research.

## Consent

Informed consent was obtained and freely given by the patient.

## Conflicts of Interest

The authors declare no conflicts of interest.

## Data Availability

Data sharing is not applicable to this article as no datasets were generated or analyzed during the current study.
